# Screening and Analysis of Potential Aquaculture Spaces for *Larimichthys crocea* in China’s Surrounding Waters Based on Environmental Temperature Suitability

**DOI:** 10.3390/biology14020205

**Published:** 2025-02-15

**Authors:** Ling Yang, Weifeng Zhou, Xuesen Cui, Yanan Lu, Qin Liu

**Affiliations:** 1 East China Sea Fisheries Research Institute, Chinese Academy of Fishery Sciences, Shanghai 200090, China; 2 College of Information Engineering, Zhejiang Ocean University, Zhoushan 316022, China

**Keywords:** *Larimichthys crocea*, deep-sea aquaculture, potential spaces, spatial analysis, China

## Abstract

This research evaluates the potential spaces of deep offshore waters for cultivating the *Larimichthys crocea*, analyzing ocean profile temperature data from 2000 to 2022 according to the species’ environmental temperature suitability. There are significant seasonal variations and differences in habitat distributions of different temperature ranges in China’s surrounding waters. The range of maximum living space obtained according to the tolerance temperature shows a trend of being larger in summer and smaller in winter; and the range of viable habitat space obtained based on the suitable and optimal temperature shows a trend of being smaller in summer and larger in winter. Broad areas meeting tolerance temperatures offer broad, yet impractical, site selection options. In contrast, areas with optimal temperatures are limited, which means the availability of ideal site locations is very restricted. Regions consistently within the 20–28 °C range are best for practical site selection. Year-round suitable areas are primarily found at depths of 30 to 90 m in the southern East China Sea and the South China Sea, particularly within the 40 to 50 m depth range. Water mass like the South China Sea Surface Water and the Kuroshio Surface Water consistently maintain suitable temperatures, making them ideal for aquaculture.

## Simple Summary

As challenges related to China’s nearshore aquaculture arise because of intensified space and environmental constraints, deep-sea aquaculture is poised to be the next frontier for aquaculture development in China. The large yellow croaker (*Larimichthys crocea*), a key economic species, is the most promising option for aquaculture in China’s deep offshore waters. This study aims to screen and analyze appropriate aquaculture spaces in China’s surrounding waters for this species, based on bio-environmental suitability and marine profile temperature data. Methodologically, by collecting ocean profile temperature data from 2000 to 2022, and integrating the environmental temperature suitability of *Larimichthys crocea* (tolerance temperatures between 9 and 30 °C, suitable temperatures of 20–28 °C, and optimal at 25 °C), this research identifies feasible areas for deep-sea farming and assesses the distribution changes across different depths, seasons, and water masses. This research result could serve as a guide to stakeholders and management of aquaculture in selecting optimal sites for cultivating the *Larimichthys crocea*, thus supporting the development of China’s aquaculture into deeper waters.

## 1. Introduction

Fisheries resources play a crucial role in the global food supply chain, particularly for developing countries, where seafood often constitutes a primary source of protein [1]. It is estimated that approximately 3 billion people rely on seafood as their main source of animal protein, thus highlighting the significant contribution of the fisheries and aquaculture sectors to the global economy [2]. China is one of the few countries in the world where aquaculture production exceeds the volume of wild catch [3]. Currently, China’s marine aquaculture primarily focuses on land-based and nearshore areas with water depths not exceeding 20 m [4]. However, these regions are significantly impacted by the development of other sectors within the socio-economic landscape, leading to severe limitations on aquaculture space and challenges such as excessive farming density, frequent disease outbreaks, and environmental degradation [5]. Consequently, in recent years, China has made substantial efforts to develop deep-sea aquaculture.

Deep-sea aquaculture refers to aquaculture activities conducted in open ocean regions, utilizing robust storm-resistant infrastructures, including steel structures or enclosed containers and automated feeding systems [6]. Typically situated beyond three nautical miles offshore and in unprotected open waters with depths ranging from 25 to 100 m, this aquaculture model has garnered significant global attention in recent years [7]. Its importance lies in alleviating pressure on nearshore aquaculture, enhancing farming efficiency and yield, and increasing environmental capacity for aquaculture [8]. Although the initial investment for deep-sea aquaculture is relatively high, the distance from densely populated human activities results in cleaner water quality, which can reduce disease occurrences and mitigate health risks associated with farming. Moreover, deep-sea farming areas generally possess higher environmental capacity, supporting larger-scale aquaculture operations that contribute to improved production efficiency and economic returns [9]. Additionally, products from deep-sea aquaculture often exhibit superior quality, meeting the market’s demand for premium seafood. This is largely due to the stable environmental conditions at greater depths, where temperature consistency and reduced human interference support healthier, more resilient species.

These benefits are evident in successful depth-specific strategies applied to various aquaculture species. For instance, in Norway, salmon farming has utilized depth preferences linked to thermal refuges and feeding behaviors to optimize cage placement, ensuring species thrive in their preferred thermal environments [10]. In Indonesia, habitat modeling for tropical groupers incorporated monthly temperature data to predict suitable breeding grounds, demonstrating how seasonal variations in temperature influence species’ habitat suitability [11]. In Australia, depth-specific models for barramundi farming aligned growth rates with the species’ thermal preferences [12], maximizing yields throughout the year. Similarly, in Chile, depth-based analyses in salmon farms have been used to reduce disease risks by identifying water layers with lower pathogen prevalence [13], further emphasizing the significance of depth in aquaculture practices. By applying a similar approach to analyze seasonal temperature and depth variations in the potential farming areas for *Larimichthys crocea* in China, valuable insights can be gained to enhance cultivation conditions and improve aquaculture sustainability.

*Larimichthys crocea*, commonly known as the Chinese yellow croaker, is one of the economically significant fish species in China’s surrounding waters, boasting high economic value. Due to its strong adaptability and favorable economic returns, it is considered an ideal candidate for deep-sea aquaculture in China [14]. Renowned in the market as “gold from the sea”, its tender flesh and delicious flavor, along with its high content of protein and essential trace elements, make it highly favored by consumers [15]. Additionally, the aquaculture and sale of *Larimichthys crocea* provide a stable source of income for coastal fishermen, contributing to local economic development. This species is classified as a warm–temperate demersal fish, typically inhabiting deeper waters at depths of up to 60 m [16]. Therefore, deep-sea aquaculture of *Larimichthys crocea* represents a new direction for development, demonstrating significant potential and application value in the deep-sea farming sector. It not only addresses the resource and environmental constraints faced by traditional aquaculture but also introduces new growth opportunities for the *Larimichthys crocea* industry. By analyzing temperature distributions across various depths within the study region, it becomes possible to identify optimal layers for growth, breeding, and feeding, thereby enhancing aquaculture efficiency and sustainability. Furthermore, incorporating depth-specific models can help mitigate environmental stresses, leading to improved health and higher yields. This approach not only strengthens the theoretical foundation of habitat suitability modeling in marine aquaculture but also emphasizes the importance of depth-specific research for effective management, promoting ecological balance and economic viability.

Developing deep-sea aquaculture is inevitably influenced and constrained by the marine environment, with ocean profile temperature being a crucial factor to consider. Additionally, variations in water masses within the coastal marine environment must be taken into account. Water masses are defined as finite or infinite bodies of water with distinct and conservative physical and chemical characteristics; those in coastal and marginal seas often exhibit more complex structures, smaller volumes, and stronger seasonal variations [17]. This study collects ocean profile temperature data of China’s surrounding waters to explore potential spaces for deep-sea aquaculture of *Larimichthys crocea* based on their tolerance and optimal temperature ranges. By utilizing temperature screening and water mass analysis, combined with seasonal changes, this study examines the environmental spatial conditions suitable for aquaculture at varying depths. Furthermore, it evaluates different aquaculture techniques suitable for *Larimichthys crocea* in deep-sea environments, aiming to enhance aquaculture efficiency and promote the sustainable development of ecological environments.

## 2. Data and Methods

### 2.1. Data Acquisition and Preprocessing

The impact of the marine environment on fisheries resources is multidimensional, involving various factors such as seasonal variations, geographical location, and seawater depth. Among these, seawater temperature is one of the most significant and direct environmental factors affecting fisheries resources. Changes in seawater temperature due to different seasons, latitudes, and depths can influence the distribution of marine biological resources. Therefore, selecting appropriate marine data sources for data acquisition and preprocessing is crucial for conducting research in fisheries oceanography.

This study utilizes data provided by the Data Center for Marine Science of the Chinese Academy of Sciences, specifically the IAP global ocean temperature 0.5° gridded dataset [18] (http://www.ocean.iap.ac.cn/pages/dataService/dataService.html?languageType=en&navAnchor=dataService, accessed on 14 February 2025). The dataset is based on a global ocean temperature dataset, which includes field observational data obtained from various instruments (such as XBT, CTD, Argo, Bottle, MBT, Glider, and mooring) in the World Ocean Database (WOD). To ensure the reliability and accuracy of the data, historical XBT data have undergone bias correction using the CH14 correction scheme proposed by the Institute of Atmospheric Physics. Additionally, spatial interpolation of the data has been performed using the improved ensemble optimal interpolation method (Mapping) suggested by the Institute of Atmospheric Physics. This interpolation method combines historical simulations from multiple models in CMIP5 with high-resolution samples to provide dynamic ensemble samples [19]. The data utilized in this study span the time range from 2000 to 2022, with seawater depth divided into 41 layers at 2000 m intervals, and are presented in a 0.5° × 0.5° grid format.

### 2.2. Study Area

*Larimichthys crocea* primarily inhabits the coastal waters of the East Asian continent [20], making it essential to investigate the relationship between environmental variables and the distribution of this species. Temperature is particularly significant; studies indicate that the tolerance temperature range for *Larimichthys crocea* is between 9 and 30 °C [21], while the suitable temperature range is 20–28 °C [22]. Within this optimal range, *Larimichthys crocea* exhibits the highest growth rate and feed conversion efficiency, alongside optimal reproductive and immune functions [23]. For this study, a comparative analysis identifies 25 °C as the optimal temperature for *Larimichthys crocea*. Although this species can survive outside the optimal range but within the tolerance range, it may experience slower growth, reduced feed conversion rates, and compromised immunity.

Furthermore, studies have found a positive correlation between the abundance of *Larimichthys crocea* and seawater depth and temperature [24]. Seawater stratification is primarily caused by temperature variations [25]. In the Chinese seas, temperature differences across different depth layers can vary significantly due to oceanic phenomena, particularly during summer and winter [26]. Additionally, the geographic location and maximum depth of various marine regions in China greatly influence the aquaculture of *Larimichthys crocea*. The Bohai Sea, a semi-enclosed body of water connected to the Yellow Sea, has an average depth of 18 m [27]. The Yellow Sea is a shallow basin in the northern part of the eastern China Sea, with an average depth of 44 m [28]. The East China Sea is a large continental shelf located between latitudes 25° and 32° N in the western Pacific [29]. The South China Sea is a semi-enclosed marine basin, with its only deep-water renewal passage located in the northeast, connecting the Luzon Strait to the northwestern Pacific [30]. The Taiwan Strait serves as a waterway linking the northern East China Sea and the southern South China Sea, situated between the Chinese mainland and Taiwan Island, with most of the strait’s depth being less than 80 m and an average depth of approximately 60 m [31].

**Figure 1:**
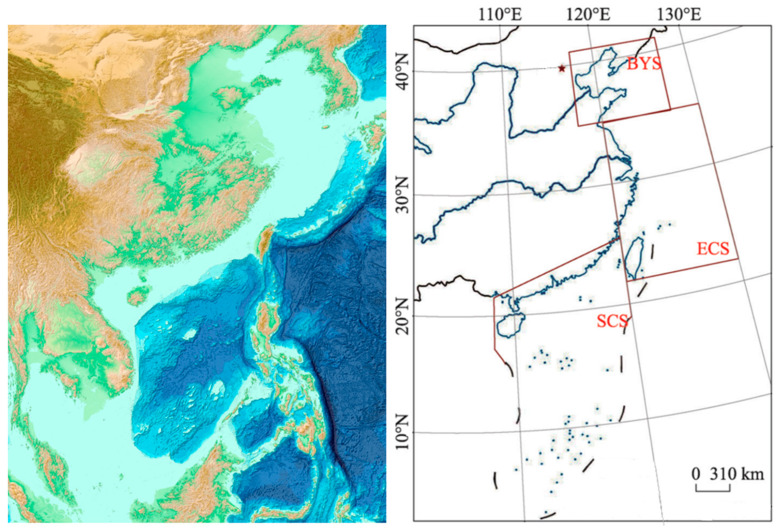
The study area of China’s surrounding waters.

The study area is set in the offshore and adjacent seas of China, covering a latitude and longitude range of 0° to 42° N and 98° to 132° E (Figure 1). The coordinates (117.5° E–127.0° E, 35.0° N–41.0° N) represent the Bohai and Yellow Seas, (120.0° E–130.0° E, 22.0° N–35.0° N) denote the East China Sea, and (108.0° E–120.0° E, 3.4° N–25.5° N) indicate the South China Sea [32].

This study conducts a layered analysis of the water masses suitable for *Larimichthys crocea* aquaculture in various depths and regions surrounding China. By comparing the accommodating spaces of different aquaculture temperature ranges, this research aims to identify the optimal aquaculture space for *Larimichthys crocea*.

### 2.3. Research Methodology and Technical Route

This study focuses on the survival environment of *Larimichthys crocea*, exploring the distribution of aquaculture spaces suitable for this species across different temperature ranges. The temporal scope of the research spans from 2000 to December 2022. First, the global ocean temperature dataset is confined to the waters surrounding China (latitude 0° to 42° N, longitude 98° E to 132° E). Second, existing research determines the temperature ranges for *Larimichthys crocea* aquaculture, including the tolerance range 9–30 °C [21], suitable range 20–28 °C [22], and the optimal temperature 25 °C. Subsequently, temperature data are cleaned to extract areas meeting the temperature conditions at various depths. Finally, the processed data undergo format conversion, presenting the analysis results through professional data visualization methods to create the most intuitive imagery.

The aquaculture space matching the temperature ranges is represented as follows:

(1) $$\{\;\left(N_{k},i,j\right)\;\left|\;temp.min\right.\leq temp_{\left(N_{k},i,j\right)}\;\leq temp.max\;\}.$$

The geographic scope of the surrounding marine areas of China is defined as follows:

(2) $$98^{\circ }\leq i\leq 132^{\circ }E,0^{\circ }\leq j\leq 42^{\circ }N.$$

Tolerance temperature range for maximum living space: $\left[{9\;}^{\circ }C,{30\;}^{\circ }C\right];$

Suitable temperature range for favorable aquaculture space: $\left[{20\;}^{\circ }C,28^{\circ }C\right];$

Optimal temperature for ideal aquaculture space: $\left[{25\;}^{\circ }C\right]$.

In the formula, temp represents temperature, i indicates longitude, j denotes latitude, $\left(i,j\right)$ refers to the corresponding geographic coordinates, k signifies water depth, and $N_{k}$ indicates the classification of water depth layers. This formula states that for each water depth layer class $N_{k}$, the recorded minimum temperature at the coordinates $\left(i,j\right)$ must be greater than or equal to the minimum temperature threshold temp.min, and the recorded maximum temperature must be less than or equal to the maximum temperature threshold temp.max. This ensures that the selected data meet the temperature requirements for the survival of *Larimichthys crocea*. During depth analysis, for the sake of mapping and expression, adjacent depth intervals are consolidated.

In this study, a global ocean temperature dataset was processed to extract latitude and longitude data specific to the Chinese sea areas and to filter suitable temperature ranges, while standardizing variable names. The netCDF format data were then converted into a CSV format that facilitates analysis, followed by comprehensive data analysis and visualization. Various libraries in the Python programming language (3.10.2) were employed to support data processing and visualization efforts. To ensure the reliability and accuracy of the dataset, a thorough data preparation process was conducted. This included identifying and excluding temperature and environmental outliers. Missing values were checked, and outlier detection was performed by focusing on the consistency of temperature values within the predefined acceptable range. Anomalous data were detected by analyzing spatial distributions based on latitude and longitude, and by applying specific temperature thresholds. NumPy (1.24.4) and Pandas (2.2.2) were utilized for efficient array and DataFrame operations, while Matplotlib (3.5.3) and Basemap (1.4.1) were used to generate diverse charts and maps. Xarray (2024.2.0) focused on managing multidimensional data, and regular expressions along with file operation libraries (re, os, and glob) assisted in data cleaning and file management. Additionally, FontProperties (3.5.3) and rcParams (3.5.3) were used to enhance chart aesthetics and font settings.

The three-dimensional visualization component employs the specialized software program Voxler (4.0.476), which can import data from various sources and create stunning 3D graphics, allowing users to creatively explore relationships within the data [33]. Its gridding capability converts discrete temperature scatter data into regular grid data, utilizing interpolation algorithms to generate continuous three-dimensional temperature fields, making the data easier to process and analyze. Coordinate or scale transformations are applied to the gridded data to facilitate comparisons of temperature data across different months within the same coordinate system. Finally, surface rendering techniques are employed to generate three-dimensional isopleth maps, illustrating the distribution and trends of temperature across different ranges in space. The Voxler software processes the temperature scatter plots for various months through gridding, transformation, and surface rendering, thereby revealing the spatial distribution differences in the survival temperatures for *Larimichthys crocea*.

**Figure 2:**
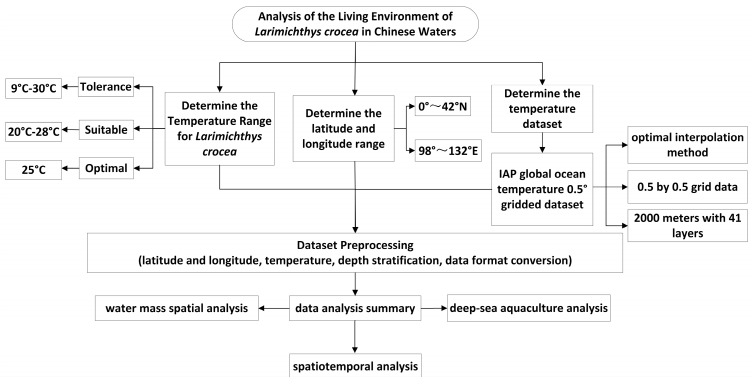
Technical flow chart of this research.

These tools significantly enhance the efficiency of data analysis and the readability of the results, as illustrated in the research route shown in Figure 2.

## 3. Screening and Analysis According to the Bio-Environmental Suitability

Generally, the tolerance temperature range for *Larimichthys crocea* is between 9 and 30 °C [21]. When water temperatures fall below or exceed this range, the feeding rate of *Larimichthys crocea* significantly declines, severely threatening their survival and potentially leading to substantial mortality and aquaculture failures. The favorable temperature range for growth is 20–28 °C [22], within which growth rate and feed efficiency are maximized, allowing for the highest growth rates of *Larimichthys crocea*.

This study analyzed the distribution of potential aquaculture spaces according to temperature ranges of environmental suitability for *Larimichthys crocea* from 2000 to 2022 in the waters surrounding China. By examining the changes in the preferred temperature range over time and depth, the aim is to provide insights for aquaculture site selection, taking into account the constraints imposed by water depth and temperature on the behavior of *Larimichthys crocea*, ultimately identifying suitable areas for aquaculture activities.

### 3.1. Screening and Analysis for Maximum Living Space According to the Tolerance Temperature

The tolerance temperature range for *Larimichthys crocea* is between 9 °C and 30 °C, which represents the maximum living space for this species in marine environments. By analyzing the temporal and spatial data from 2000 to 2022 within this 9–30 °C range, this study aims to identify the optimal conditions for *Larimichthys crocea* aquaculture.

#### 3.1.1. Seasonal Variations in the Size of the Maximum Living Space

**Figure 3:**
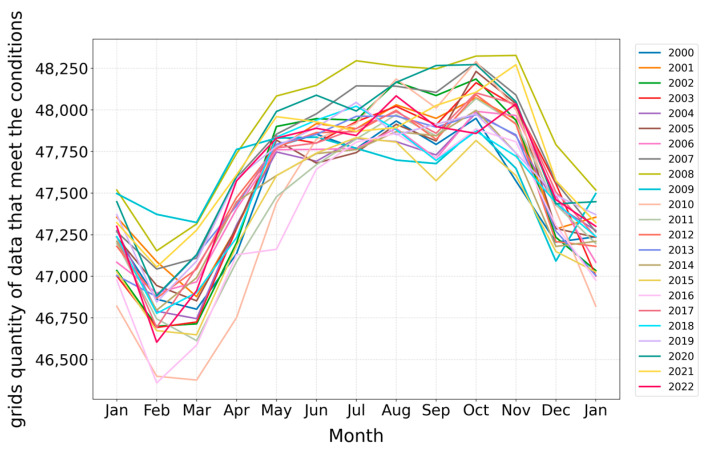
Inter-annual and seasonal changes in grid points’ numbers under the condition of the biological tolerance temperature from 2000 to 2022.

This study employs line graphs to analyze the changes in the size of the maximum living space (determined by the tolerance temperature range of 9–30 °C) over time. Grid points of ocean environmental data that meet the tolerance temperature range were selected and statistically analyzed for the years 2000 to 2022. The entire dataset comprises 13,134,213 grid points, with monthly data ranging from 45,000 to 49,000 points. Utilizing these data, this study conducts an in-depth analysis of the trends in tolerance range variations across different months, using lines of various colors to distinguish between years, as shown in Figure 3.

Marine temperatures are typically influenced by seasonal variations. Analysis of the trends in the number of qualifying ocean environmental data points indicates a consistent pattern of temperature changes across different years. The tolerance temperature range for *Larimichthys crocea* generally reaches its lowest point in February or March. From March to June, as seasonal temperatures increase, the tolerance temperature range exhibits a rapid growth trend. During the summer months of June to August, this range continues to expand, peaking around October. Following October, as winter approaches, the tolerance temperature range declines sharply. The decrease observed in September, as illustrated in Figure 3, may be attributed to the gradual reduction in solar radiation after summer. Due to the ocean’s high heat capacity, temperature changes in deeper waters tend to lag, which may lead to increased mixing between surface and deep waters in October, resulting in a temporary rise in surface temperatures.

The inter-annual and seasonal variations in the number of ocean environmental data points that meet the tolerance temperature conditions indicate that the maximum living space range for *Larimichthys crocea*, constrained by its tolerance temperature, occurs during the summer months in the waters surrounding China.

#### 3.1.2. Variation in the Maximum Living Space with Depth

**Figure 4:**
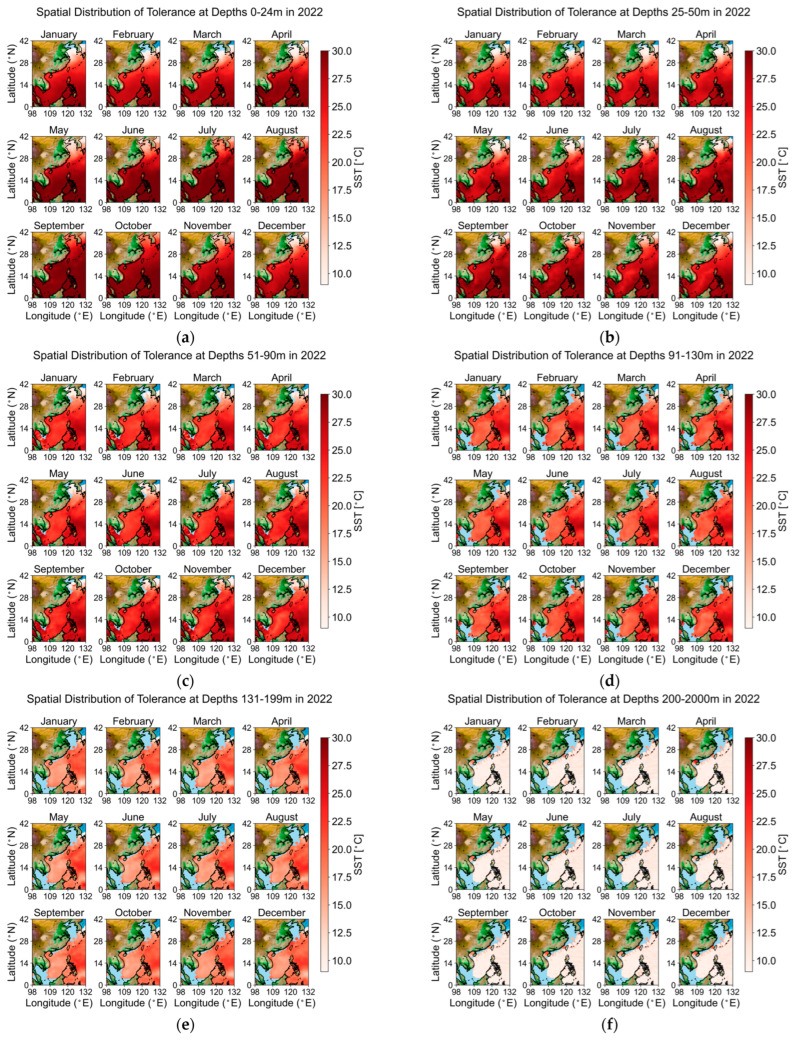
In 2022 Geographic Map of Tolerance Temperature by Depth. This figure illustrates the monthly spatial distribution of temperature tolerance across various ocean depths throughout 2022: (**a**) 0–24 m depth; (**b**) 25–50 m depth; (**c**) 51–90 m depth; (**d**) 91–130 m depth; (**e**) 131–199 m depth; (**f**) 200–2000 m depth.

To facilitate mapping and analytical expression, depth intervals were categorized into six groups (1–24 m, 25–50 m, 51–90 m, 91–130 m, 131–199 m, and 200 m and above) on a monthly basis. Using the temperature data from 2022 as an example, the regions that meet the tolerance temperature conditions for *Larimichthys crocea* are shown in red, with darker shades indicating higher temperatures, while blue areas represent regions that do not meet the temperature range. The results are illustrated in Figure 4.

Comparison reveals that, generally, in waters shallower than 90 m, all areas in China, except for certain regions in the Bohai Sea during winter, fall within the tolerance temperature range for *Larimichthys crocea*. However, when the depth exceeds 90 m, only a few areas in the East China Sea and the South China Sea remain within this tolerance range, with temperatures gradually decreasing as depth increases. Beyond 200 m, only certain regions in the South China Sea are included, primarily due to the average depth of the East China Sea being around 300 m, while the continental shelf depth does not exceed 200 m.

### 3.2. Screening and Analysis for Favorable Aquaculture Space According to the Suitable Temperature

The suitable growth temperature range for *Larimichthys crocea* is 20–28 °C. Within this temperature range, the survival environment is conducive to its growth. Suitable temperature conditions not only promote rapid growth but also positively influence healthy development throughout its life cycle. Therefore, areas within this temperature range can be considered as relatively favorable aquaculture space for *Larimichthys crocea* in marine environments. It is evident that the range of suitable temperatures is smaller than that of the maximum temperatures.

#### 3.2.1. Seasonal Variations in the Size of the Favorable Aquaculture Space

**Figure 5:**
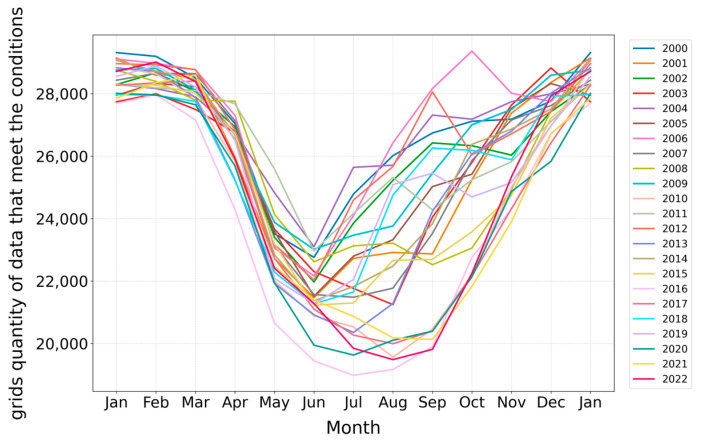
Inter-annual and seasonal changes in grid points’ numbers under the condition of the biological suitable temperature from 2000 to 2022.

This study employs line graphs to analyze the variation in the size of favorable aquaculture space, determined based on the suitable temperature range of 20 °C to 28 °C, over time. We filtered and statistically assessed the number of oceanographic data grid points meeting the suitable temperature range from 2000 to 2022. The dataset comprises 6,983,653 grid points, with monthly data ranging between 20,000 and 30,000 grid points. Utilizing these data, we conducted an in-depth analysis of the trends in suitable ranges across different months, distinguishing years with lines of varying colors, as illustrated in Figure 5.

The analysis of the trend in the number of oceanographic data grid points reveals that the overall temperature variation trends are consistent across different years. However, the size change pattern of favorable aquaculture space is completely different from that of the maximum living space, varying distinctly by season throughout the year. The range of favorable aquaculture space is widest in January and February during winter. From February to June, there is a rapid decrease in suitable deep-sea aquaculture space as the months progress. Between June and November, significant inter-annual fluctuations occur, with the least favorable aquaculture space during the summer months of June to August. Conversely, from September to November, as seawater temperatures decrease, the suitable areas increase, reaching their maximum in winter.

#### 3.2.2. Variations in the Favorable Aquaculture Space with Depth

**Figure 6:**
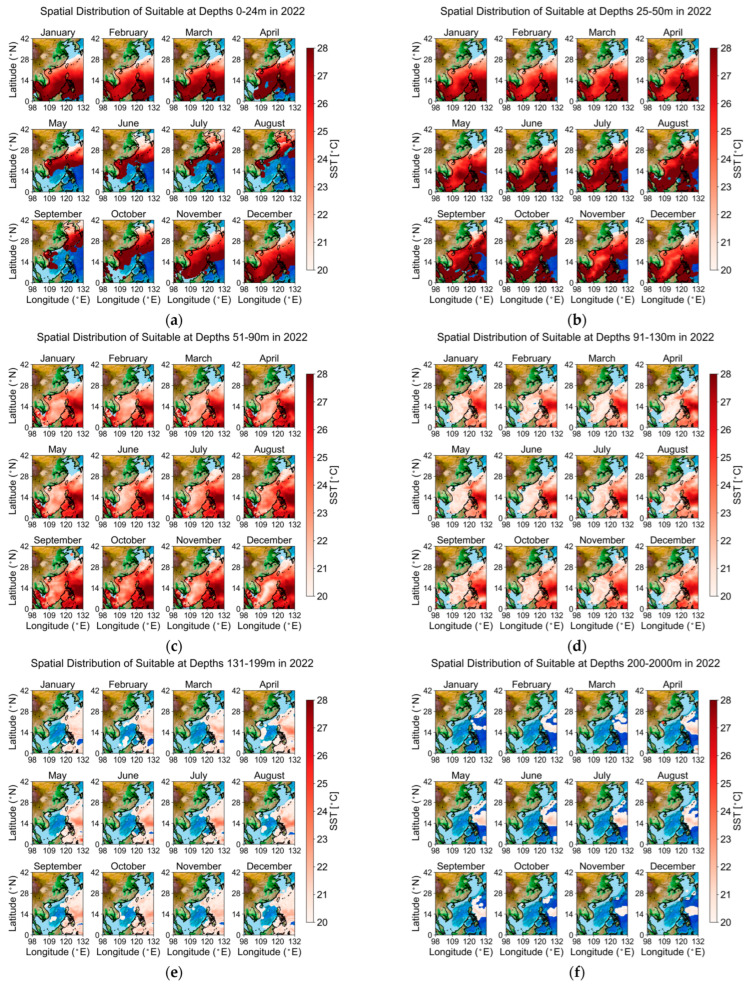
In 2022 Geographic Map of Suitable Temperature by Depth. This figure illustrates the monthly spatial distribution of temperature suitable across various ocean depths throughout 2022: (**a**) 0–24 m depth; (**b**) 25–50 m depth; (**c**) 51–90 m depth; (**d**) 91–130 m depth; (**e**) 131–199 m depth; (**f**) 200–2000 m depth.

To facilitate mapping and analytical representation, depth ranges were categorized into six classes on a monthly basis: 1–24 m, 25–50 m, 51–90 m, 91–130 m, 131–199 m, and 200 m and deeper. Using the temperature data from 2022 as an example, this study presents the results by month and depth categories, as shown in Figure 6. Areas within the suitable temperature range for *Larimichthys crocea* are indicated in red, with deeper shades representing higher temperatures. The blue regions denote areas that do not meet the temperature criteria.

Due to the relatively shallow waters of the Yellow Sea and Bohai Sea, the summer season is favorable for aquaculture in shallow marine areas below 25 m. However, these regions lack the appropriate water temperatures for *Larimichthys crocea* in winter. Conversely, in the South China Sea, shallow areas are favorable for aquaculture during winter, but high temperatures in summer render them unsuitable for this species. This creates a stark contrast in aquaculture conditions between the northern and southern seas across different seasons. Therefore, the suitable space for deep-sea farming primarily encompasses the East China Sea, South China Sea, and Taiwan Strait. In the depth range of 25 to 50 m, the water temperatures conducive to survival are mainly concentrated in the East and South China Seas, with minimal monthly variations, and winter temperatures are generally lower than in summer. At depths of 51 to 90 m, suitable temperatures for deep-sea aquaculture of this fish decrease in these regions. Beyond 90 to 130 m, temperatures typically fall below 25 °C, and depths greater than 130 m are generally unsuitable for deep-sea aquaculture.

### 3.3. Screening and Analysis of Ideal Aquaculture Space According to the Optimal Temperature

This section explores the variation in optimal aquaculture space over time and across different regions, based on the optimal temperature range for *Larimichthys crocea*, which is 25 °C. Within this temperature range, the conditions are ideal for aquaculture. It is evident that the optimal aquaculture space is smaller than the favorable aquaculture space.

#### 3.3.1. Seasonal Variations in the Size of the Ideal Aquaculture Space

**Figure 7:**
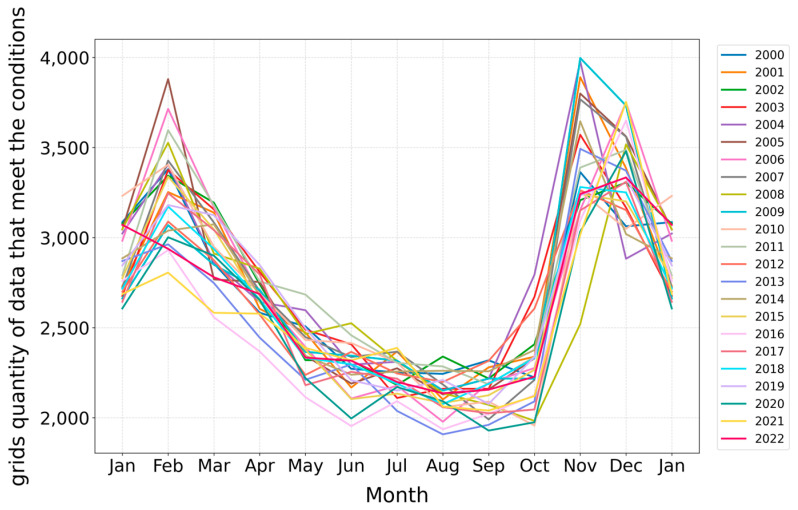
Inter-annual and seasonal changes in grid points’ numbers under the condition of the biological optimal temperature range from 2000 to 2022.

This study employs line graphs to analyze the variation in the size of ideal aquaculture space, determined based on the optimal temperature range of 25 °C, over time. We filtered and statistically assessed the number of oceanographic data grid points that meet the tolerance temperature range from 2000 to 2022. The dataset comprises a total of 733,577 grid point entries, with monthly data ranging between 2000 and 3500 grid points. Utilizing these data, we conducted an in-depth analysis of the trends in optimal ranges across different months, distinguishing years with lines of varying colors, as illustrated in Figure 7.

Analysis of the trend in the number of ocean data grid points indicates that the overall temperature variation trends are consistent across different years. However, the seasonal changes in the range of ideal aquaculture space differ from those of the favorable aquaculture space. Notably, both exhibit minimal ideal aquaculture space during summer; however, this period lasts nearly four months. After October, the ideal aquaculture space increases rapidly, with peaks observed in November and February. Following February, there is a gradual decrease in optimal space leading into June, reflecting the seasonal dynamics that influence growth conditions.

#### 3.3.2. Variation in Ideal Aquaculture Space with Depth

**Figure 8:**
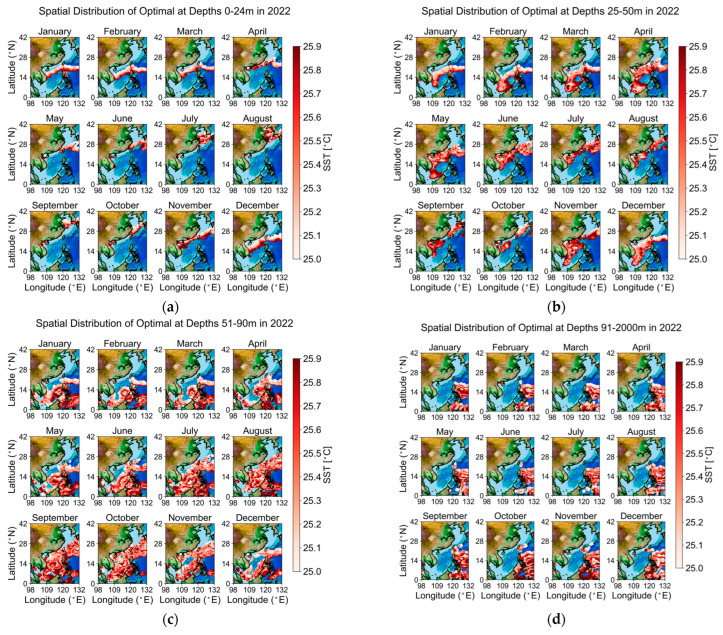
In 2022 Geographic Map of Optimal Temperature by Depth. This figure illustrates the monthly spatial distribution of temperature optimal across various ocean depths throughout 2022: (**a**) 0–24 m depth; (**b**) 25–50 m depth; (**c**) 51–90 m depth; (**d**) 91–2000 m depth.

This section further explores the distribution of ideal aquaculture space, defined by the optimal temperature range for *Larimichthys crocea*, which is 25 °C. To facilitate mapping and analytical representation, depth ranges are categorized into four classes on a monthly basis: 1–24 m, 25–50 m, 51–90 m, and greater than 90 m. Using the temperature data from 2022 as an example, the results are illustrated by month and depth categories in Figure 8. Areas within the optimal temperature range for *Larimichthys crocea* are indicated in red, with deeper shades representing higher temperatures, while blue regions denote areas that do not meet the temperature criteria. The analysis reveals a clear seasonal variation in the spatial distribution of ideal aquaculture space, with significant fluctuations observed.

In the 1–24 m shallow water zone, ideal aquaculture areas are predominantly located in the South China Sea and East China Sea during winter, while ideal regions in summer are found in the Bohai Sea and Yellow Sea. As the season transitions from summer to winter, the temperature distribution shifts southward, with significantly less aquaculture space compared to deep-sea farming. In the 25–50 m range, the optimal temperatures in winter are mainly found in the South China Sea, while in summer, they are more prevalent in the East China Sea. With increasing depth, a trend is observed where the optimal temperature zones for *Larimichthys crocea* migrate from the East China Sea to the South China Sea. In the 51–90 m range, optimal temperatures are primarily concentrated in the South China Sea, with summer distributions extending to a few areas in the East China Sea, while winter suitability is limited to a small number of regions in the South China Sea. Beyond 90 m, the majority of optimal temperature ranges fall outside of Chinese territorial waters.

### 3.4. Screening for the Areas That Are Always in the Suitable Temperature Range

For aquaculture practitioners, identifying regions with consistently suitable temperatures can aid in selecting favorable aquaculture locations, thereby enhancing aquaculture conditions and improving farming efficiency. Therefore, this study also examines areas that remained within the suitable temperature range 20–28 °C throughout the entire dataset from 2000 to 2022. By filtering the data, a total of 7598 grid points were identified that meet this criterion.

**Figure 9:**
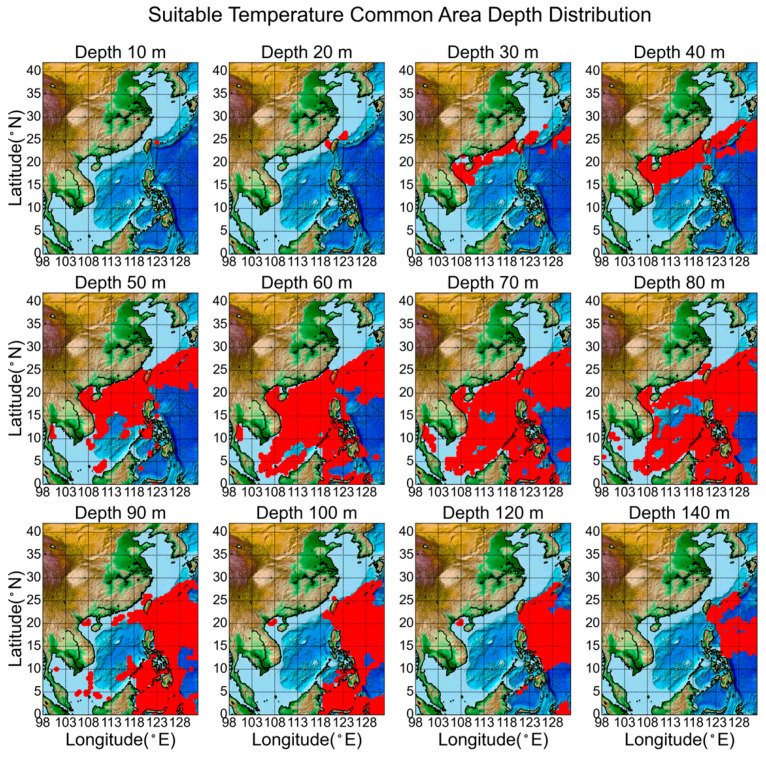
Common map of areas with suitable temperatures by depth from 2000 to 2022.

The results are illustrated in Figure 9, where the red regions indicate areas that have remained within the suitable temperature range for *Larimichthys crocea* over a span of 22 years. Different subplots represent varying depths. From an environmental perspective, these regions consistently provide favorable conditions for the growth of this species, making them ideal environments for breeding and development.

Figure 9 reveals that there are almost no areas at the surface (0–20 m) consistently within the suitable temperature range for *Larimichthys crocea*. This is attributed to the shallower water layers, which experience significant temperature fluctuations influenced by seasonal and weather changes. Starting at a depth of 30 m, the number of areas consistently suitable for this species increases, but it decreases rapidly at 90 m. This indicates that the suitable temperature range for *Larimichthys crocea* is primarily found between 30 and 90 m, where water temperatures are more stable and less affected by surface climate variations. This suggests that from a marine environmental perspective, deep-sea aquaculture can effectively leverage the favorable temperature conditions of the ocean.

Spatially, these regions are predominantly located in the southeastern East China Sea and the South China Sea. At depths of 30–50 m, the optimal zones are primarily concentrated in the southeastern East China Sea. At a depth of 60 m, the distribution is the widest, while the 60–80 m range is extensively found in the South China Sea. However, the range contracts rapidly at depths exceeding 90 m. Given that deeper waters typically incur higher potential engineering costs, the distribution of areas consistently within the suitable temperature range at depths of 40–50 m may provide more valuable insights for aquaculture site selection. Different forms of deep-sea farming can be considered in various regions based on these findings.

### 3.5. Three-Dimensional Visualization of Aquaculture Space

Figure 4, Figure 6, and Figure 8 display the marine environment suitable for the aquaculture of *Larimichthys crocea* at different depths, using discontinuous slices. This section employs 3D visualization to directly show the distribution of temperature ranges suitable for *Larimichthys crocea* in the seas surrounding China at specific times. This study utilizes Voxler software to render three-dimensional images of the aquaculture space within suitable 20–28 °C and optimal 25 °C temperature conditions, comparing the results from different months in 2022.

**Figure 10:**
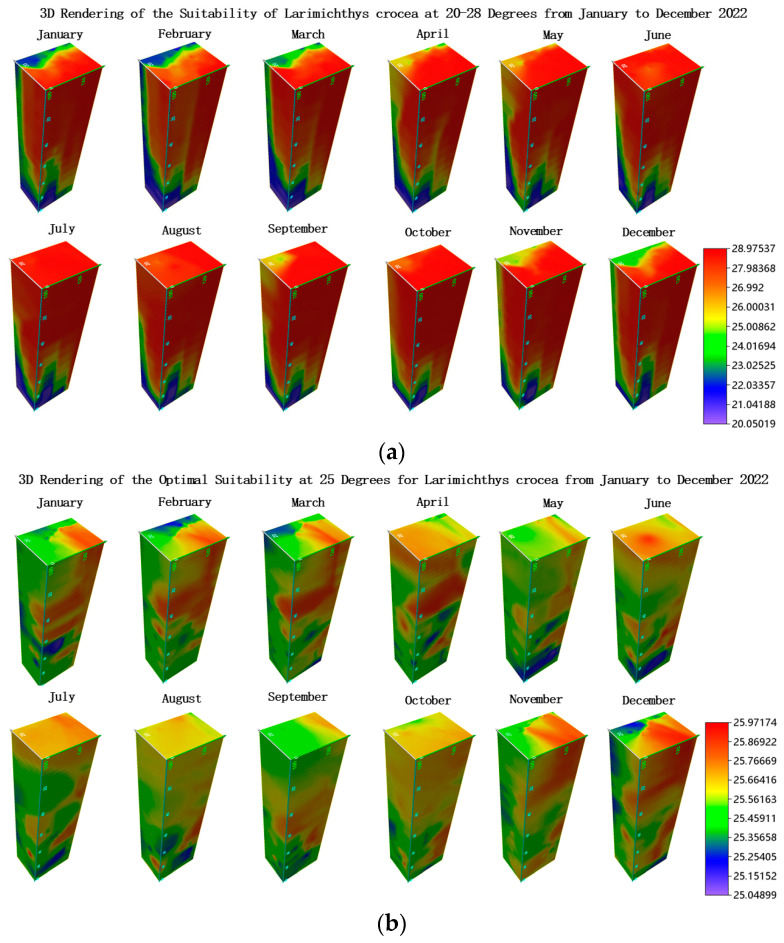
Comparative diagram of suitable and optimal spatial conditions by month for 2022: (**a**) suitable conditions; (**b**) optimal conditions.

As shown in Figure 10, the 3D renderings for the months of January through December 2022 represent the optimal temperature of 25 °C and the suitable range of 20–28 °C. The model’s three axes correspond to longitude, latitude, and water depth, where depth increases downward, and different colors represent temperatures. The color transitions from deep red to blue signify a shift from higher to lower temperatures. This color variation presents a vertical stratification in the 3D plot, indicating that while shallower waters are warmer, deeper layers are cooler, with temperatures decreasing as depth increases. However, the temperature distribution in the subsurface layers varies significantly by geographic location. The 3D spatial diagram in Figure 10 and Supplementary Video S1 effectively illustrates the temperature differences at various depths and geographic locations. Therefore, when specific sites are considered as potential locations for aquaculture facilities, the variation in water temperature at different depths over time at these sites merits careful consideration.

### 3.6. Seasonal Temperature Analysis and Clustering for Favorable Aquaculture Space of Larimichthys crocea

Seasonal temperature analysis is crucial for optimizing aquaculture site selection, especially for species like *Larimichthys crocea* in the waters surrounding China. By segmenting environmental temperature data into quarterly averages, specific temperature ranges conducive to improved habitat conditions can be identified. This approach allows for a deeper understanding of how temperature fluctuations influence the spatial distribution of suitable farming areas throughout the year.

**Figure 11:**
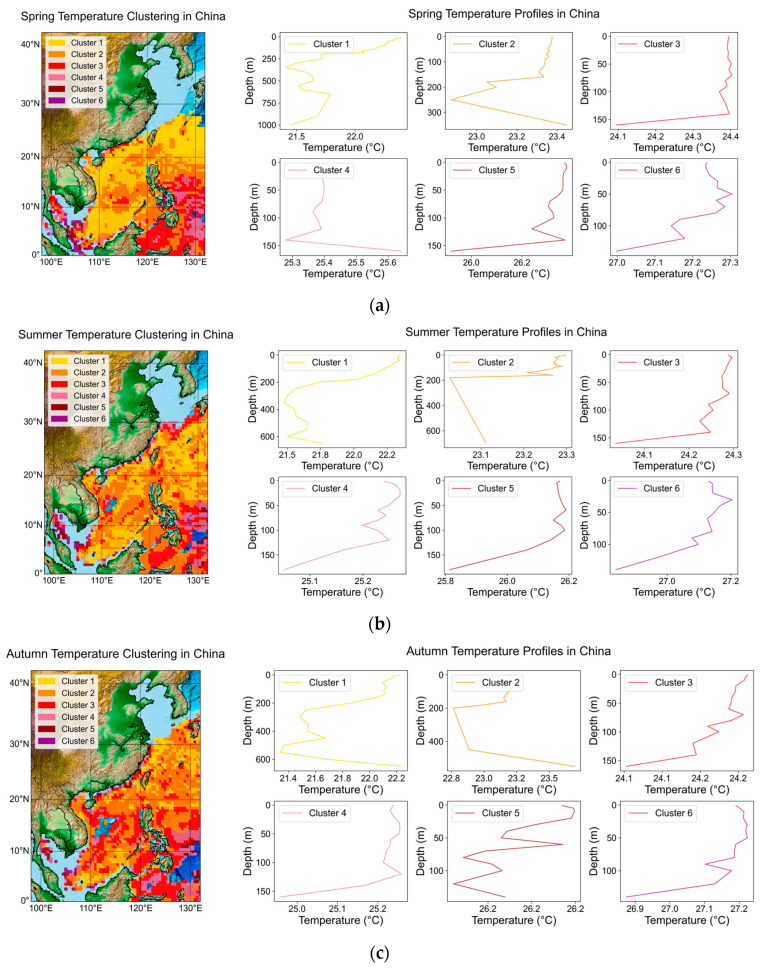

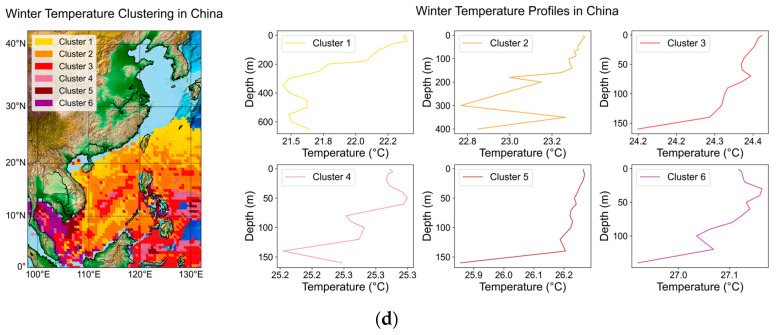
Seasonal average temperature clustering and profile analysis in China’s adjacent seas. This figure displays the spatial distribution of six K-Means clustering categories for average temperatures in different seasons from 2000 to 2022 in the seas adjacent to China: (**a**) spring; (**b**) summer; (**c**) autumn; (**d**) winter.

To calculate the average temperature for each seasonal period, we used temperature data from 2000 to 2022 across consistent latitude, longitude, and depth intervals, focusing on the 20–28 °C range, which is suitable for favorable aquaculture space. The temperature data were categorized into four distinct seasonal groups [34]: Spring (March, April, May), Summer (June, July, August), Autumn (September, October, November), and Winter (December, January, February). For each of these seasons, we calculated the average temperature by taking the mean of all available temperature readings corresponding to each specific location (latitude, longitude) and depth category for the given months. This study employs the K-Means clustering algorithm to categorize the data into six distinct clusters based on the average temperatures of different seasons. Each cluster is represented by a unique color corresponding to a specific temperature range. The average curves are presented in the profile plots, which represent the typical temperature variation trends of each cluster at different depths. Separate subplots are created for each of the six clusters across different seasons to demonstrate these trends. The clustering and profile results are illustrated in Figure 11.

In spring, a moderate thermal gradient is observed, with cooler temperatures in the northern waters transitioning to warmer conditions in the south. Summer, in contrast, features a narrower temperature range, with the highest temperatures concentrated in the southern and central regions. Autumn shows a broad distribution of cooler temperatures, particularly in northern areas, marking the onset of seasonal cooling. Winter exhibits the widest spread of the coldest temperature clusters, highlighting the significant cooling effect of seasonal air masses. These patterns reflect clear seasonal variations in sea surface temperatures, consistent with expected seasonal dynamics: winter presents the coldest and least hospitable conditions, with minimal areas suitable for marine life; spring offers moderate temperatures; summer is characterized by the highest temperatures; and autumn provides a broader range of suitable temperatures. The observed thermal gradients are essential for understanding the effects of seasonal changes on marine biodiversity, fisheries, and climate interactions in these regions.

## 4. Discussion

### 4.1. Joint Analysis of Aquaculture Space and Water Masses for Larimichthys crocea

Water masses are defined as bodies of water with a specific volume that possess relatively stable physicochemical characteristics distinct from the surrounding waters [35]. Existing research categorizes the water masses in the seas surrounding China into several types: the Bohai, Yellow Sea, and East China Sea water masses; the South China Sea water mass; and the Taiwan Strait water mass. These regional water masses contribute to the diversity of marine environments in Chinese waters, significantly impacting the local climate, ecology, and fisheries [34]. The stability of these water masses is crucial in selecting environments for deep-sea aquaculture, as their location and characteristics significantly influence offshore aquaculture [36]. This section compares the distribution of water masses across China during winter (December to February) and summer (June to August) with the aquaculture space of *Larimichthys crocea*.

Different water masses and temperature ranges are shown in Table A1 of Appendix A. The water masses of the Bohai, Yellow Sea, and East China Sea are geographically adjacent, and are therefore often considered collectively in analyses, divided into 16 main water masses [37]. The South China Sea, a semi-enclosed marginal sea of the northwest Pacific, primarily connects with the Pacific Ocean through the Luzon Strait [38]. It is divided into 10 main water masses. The water masses in the Taiwan Strait, due to the typically limited observational coverage of the entire strait and the influence of the Taiwan Warm Current and monsoons, experience significant temperature variations [39]. These water masses are divided into nine main types [34]. And the following analysis and abbreviated presentation concerning water masses are also referred to [34]. During winter and summer, the water masses in these seas exhibit different distribution characteristics. A comparison reveals that in winter, although the temperatures of the YE, Es, E, EK, and KM water masses in the Bohai, Yellow, and East China Seas fall within the tolerance range for *Larimichthys crocea*, they are below the suitable temperature range for aquaculture; hence, they are unsuitable for cultivating this species. Conversely, in summer, except for the Yc, KI, and KD water masses, most water masses exceed the optimal temperature of 25 °C for *Larimichthys crocea*. Only the temperature of the Ys water mass is ideal for aquaculture.

In the South China Sea, during winter, the temperatures of the I, D, and B water masses fall below the tolerance level for *Larimichthys crocea*, while the temperatures of other water masses are within the suitable range, with the M and KS water masses being particularly suitable for aquaculture. Although no water mass in summer exactly meets the optimal temperature standards, the temperatures of the M, US, UI, UP, and KS water masses still fall within the tolerance range for *Larimichthys crocea*, making them viable for consideration in aquaculture.

In the Taiwan Strait, water masses generally maintain higher winter temperatures compared to those in the Bohai, Yellow, and East China Seas, and consistently fall within the tolerance range for *Larimichthys crocea*. Among these, the KBW and SB-KBW water masses have winter temperatures most suitable for the growth of *Larimichthys crocea*. In summer, the temperature of the MZCW water mass is most suitable for aquaculture, followed closely by the UW and MCCW water masses, whose temperatures also fall within the suitable range for *Larimichthys crocea*. The temperatures of other Taiwan Strait water masses also meet the tolerance conditions for *Larimichthys crocea*.

Comprehensive analysis indicates that during winter, the temperatures of most water masses are close to or below the minimum tolerance temperature of 9 °C for *Larimichthys crocea*, rendering them unsuitable for aquaculture. However, in the East China Sea, the Taiwan Strait, and the South China Sea, the winter temperatures of water masses are generally higher. For instance, the Kuroshio Surface Water Mass (KS) in the East China Sea and the Surface (S) and Mixed (M) water masses in the South China Sea reach temperatures within the suitable range for *Larimichthys crocea*. In contrast, the situation is significantly different in summer, when the temperatures of most water masses are within the suitable range for aquaculture of *Larimichthys crocea*, except for the cooler temperatures in deep water masses. Thus, summer is more suitable for the aquaculture of *Larimichthys crocea* than winter. Moreover, the S in the South China Sea and the KS maintain temperatures within the suitable range for *Larimichthys crocea* aquaculture throughout the year.

### 4.2. Exploration of Deep Offshore Aquaculture Methods for Larimichthys crocea

Current deep offshore aquaculture methods primarily include offshore enclosures, stable cages, suspended net pens, submersible net pens, and semi-submersible net pens, among others [40]. Each of these systems features distinct characteristics in terms of water depth range and structural flexibility, making them suitable for various aquaculture needs and environmental conditions. Offshore enclosures are commonly used in the archipelagic areas of China and are constructed from steel, concrete, or copper alloy nets, offering low construction costs but limited structural flexibility, suitable for water depths of 5 to 20 m [41]. Stable cages, anchored by piles or a central column, are suitable for water depths of 5 to 30 m; they are easy to operate but offer limited flexibility [40]. Suspended net pens, which hang in the water, are suitable for depths of 20 to 100 m, utilizing ocean currents to provide oxygen and food [42]. Submersible net pens submerge the facility underwater to take advantage of stable temperatures and low light conditions, suitable for depths of 50 to 200 m [43]. Semi-submersible net pens, which can be ballasted or de-ballasted to adjust to different depths, are suitable for water depths of 30 to 150 m and are among the most popular infrastructures in global deep-sea aquaculture. Closed systems, including aquaculture vessels and sea-based grow-out containers, are suitable for various depths, though the specific applicable depths depend on the equipment design [44].

Water temperature and depth are critical factors in determining the success of aquaculture. The deep offshore aquaculture strategy for *Larimichthys crocea* should be based on thorough environmental analysis and the selection of appropriate aquaculture technologies. Matching different deep offshore aquaculture methods with suitable temperature ranges is essential to identify the most effective aquaculture approach [45]. By selecting suitable aquaculture systems and adjusting the aquaculture areas in a timely manner, it is possible to maximize aquaculture efficiency while protecting the marine ecological environment.

Submersible net pens, which can be submerged to depths of 50 to 200 m [46], are particularly suitable in certain areas of the South China Sea and East China Sea where deeper water temperatures are relatively stable, helping to maintain the temperature range needed for cultivating *Larimichthys crocea*. Semi-submersible net pens offer higher flexibility and can be adjusted to different environmental conditions, making it possible to find suitable water layers for aquaculture even in seasons with significant temperature fluctuations [47]. These cages are typically set at depths of 30 to 150 m and are suitable for use in a broader area of the East China Sea and South China Sea.

The application of deep offshore aquaculture technology can effectively mitigate the challenges brought by temperature fluctuations in coastal waters, providing a more stable environment for aquaculture. This is crucial for enhancing aquaculture efficiency and ensuring the sustainability of *Larimichthys crocea* aquaculture. Therefore, it is feasible to select the appropriate water layer for cultivating *Larimichthys crocea* by analyzing different water masses and considering temperature and seasonal changes. Additionally, deep offshore aquaculture also helps drive the transformation and upgrading of the aquaculture industry, promoting its sustainable development. Thus, future efforts could focus on enhancing research and development in deep offshore aquaculture technologies and innovating management practices to pave new paths and opportunities for the development of the *Larimichthys crocea* aquaculture industry.

### 4.3. International Relevance and Future Prospects for Larimichthys crocea Aquaculture

This study, through the analysis of temperature data, provides a theoretical basis for the site selection of *Larimichthys crocea* aquaculture in Chinese sea areas, considering various months and depths. The findings offer theoretical references to improve operational efficiency, sustainability, and productivity. Furthermore, similar studies conducted internationally support the broader applicability of these findings, particularly in site optimization, environmental management improvements, and informing policy decisions.

In Norway, the integration of temperature-based site selection methods has been a successful strategy for salmon farming [10]. By utilizing thermal habitat models, Norway has effectively identified optimal aquaculture sites that align with species-specific temperature requirements. Japan offers a valuable example of integrating environmental data into regional aquaculture planning, which has contributed to the development of effective coastal farming strategies. By employing temperature variability models to mitigate the risk of heat stress during peak summer months, scientific findings have been directly incorporated into regional zoning policies [48], particularly for high-value species such as amberjack. In addition, Chile’s use of thermal stability data to reduce disease risks in salmon farming exemplifies how temperature models can improve both health management and production cycles [13]. In Australia, real-time environmental monitoring has supported sustainable farming practices in barramundi farming, demonstrating how ongoing data collection can optimize production and reduce environmental stress [12]. These international cases underscore the potential benefits of incorporating temperature-based models into China’s aquaculture policies and practices, helping to mitigate risks, optimize production cycles, and ensure the long-term sustainability of aquaculture operations.

By comparing the identified aquaculture zones with successful offshore aquaculture practices in countries such as Norway and Australia, this study suggests that *Larimichthys crocea* farming in China could benefit from adopting similar temperature-based site selection strategies. A region-specific approach to zoning would enable China to ensure the sustainable expansion of aquaculture while effectively mitigating environmental risks. The integration of temperature suitability models could also inform policy frameworks in China, as demonstrated by the successful implementation of similar models in Norway and Japan. For instance, in Norway, thermal stability data have been incorporated into aquaculture policies to improve site selection and enhance the sustainability of salmon farming [49]. By adapting Norway’s temperature-based site selection methods, China could improve site identification and zoning for *Larimichthys crocea* farming, ensuring that farming zones are aligned with the species’ thermal preferences. In Japan, models of temperature variability have been used to reduce heat stress and optimize production cycles. Japan’s integration of scientific data into coastal planning could inspire China to incorporate temperature and environmental data into regional zoning efforts, further enhancing sustainability and operational efficiency [50].

In addition to the technical and policy implications, the role of collaboration in implementing the findings of this study is essential. Public–private partnerships could be established to fund and develop deep-sea aquaculture infrastructure in the identified optimal zones. These partnerships would combine the resources and expertise of the public sector, which typically provides funding and regulatory support, with the private sector, which drives innovation and operational execution. Such collaborations would facilitate the development of necessary offshore infrastructure, ensuring that selected zones are equipped with the technologies required for sustainable aquaculture operations.

Joint research initiatives involving academic institutions, government bodies, and industry stakeholders could facilitate the development of data-sharing platforms, enabling real-time monitoring and adaptive management strategies. Through collaboration, stakeholders can refine predictive models, enhance temperature and depth monitoring systems, and exchange best practices. This collective effort will be crucial for ensuring aquaculture systems remain adaptable and resilient, responding effectively to changing environmental conditions. Furthermore, international collaborations, such as participation in global aquaculture conferences or consortia, would promote knowledge exchange and the adoption of best practices suited to local contexts. These collaborations could also facilitate the sharing of technical expertise, regulatory frameworks, and sustainability models, which could directly inform the development of China’s aquaculture policies and practices. Engaging with international partners would help China align its strategies with global trends, ensuring the aquaculture industry remains competitive, sustainable, and innovative.

Looking ahead, future research in aquaculture site selection should expand the scope of environmental factors beyond the temperature-based models emphasized in this study. While temperature remains a critical factor influencing species distribution, incorporating additional environmental variables—such as salinity and hydrodynamic flow patterns—could offer a more comprehensive understanding of site suitability. For instance, salinity plays a crucial role in the health and growth of various aquaculture species, and its interaction with temperature may further influence species distribution. Similarly, hydrodynamic factors like water circulation and wave action are vital for nutrient distribution and waste management, both essential for maintaining optimal farming conditions.

Several international examples highlight the potential of integrating additional environmental variables into aquaculture management. In South Korea, studies on seaweed farming have successfully incorporated hydrodynamic and temperature models to optimize yields [51]. Similarly, in New Zealand, regional temperature and salinity models have been used to expand shellfish farming into nontraditional areas, identifying regions previously considered unsuitable [52]. This success exemplifies how environmental data can guide the strategic expansion of aquaculture zones. Integrating these factors with the temperature-based models developed in this study would enhance the robustness of tools for site selection, ensuring greater sustainability and efficiency in aquaculture practices. For China, this approach could lead to the expansion of aquaculture operations into offshore and deeper water zones, ensuring that new farming areas are both environmentally suitable and economically viable.

Additionally, future studies should consider emerging anthropogenic stressors, such as pollution and microplastic contamination, which are increasingly significant concerns in aquaculture regions. Remote sensing data could be used to monitor pollution levels, while bioindicator species could assess microplastic concentrations. Integrating such data with environmental models would help identify pollution hotspots, evaluate their impact on aquaculture species, and develop mitigation strategies. Advanced modeling tools could also simulate the long-term impacts of these stressors on aquaculture sustainability, providing actionable insights for industry stakeholders and policymakers. This will be particularly crucial for species like *Larimichthys crocea* and other economically important species, which may be particularly vulnerable to these emerging threats. By addressing these challenges, future research can further enhance the resilience of aquaculture systems and promote the long-term sustainability of the industry.

## 5. Conclusions

The *Larimichthys crocea* is a species sensitive to temperature fluctuations, and its habitat is closely related to water temperature. This study focuses on the environmental adaptation characteristics of the species, specifically its temperature adaptation features, to explore the potential aquaculture space for *Larimichthys crocea* in the seas around China, and to understand how its distribution changes with seasons and depths. These temperature conditions include an optimal temperature of 25 °C, a suitable temperature range of 20–28 °C, and a tolerance temperature range of 9–30 °C. This study found that the living space under these temperature conditions showed significant seasonal variations and differences. Among them, the maximum living space determined based on the tolerance temperature had the widest spatial distribution, but its range provides limited guidance for deep-sea farming site selection. The area based on the optimal temperature range had a very low proportion, indicating that if only the ideal temperature conditions are considered, the aquaculture area for *Larimichthys crocea* is extremely limited. Thus, the region consistently within the suitable temperature range (20–28 °C) from 2000 to 2022 is of the greatest reference value for deep-sea farming practices for *Larimichthys crocea*.

The findings of this study are expected to provide useful references especially in the aspect of site selection for deep-sea farming of *Larimichthys croce* and contribute to the sustainable development of the aquaculture industry. This indicates that when designing aquaculture strategies for *Larimichthys crocea*, seasonal temperature changes and the specific temperature conditions of different water masses must be taken into account. With the development of aquaculture technologies, deep-sea farming systems, such as submerged and semi-submerged cages, provide new possibilities for *Larimichthys crocea* aquaculture. These systems allow farming in deeper waters, taking advantage of more stable temperature conditions and avoiding the impact of seasonal temperature fluctuations in coastal areas.

## Data Availability

Data are available upon request from the authors.

## Supplementary Materials

The following supporting information can be downloaded at: https://www.mdpi.com/article/10.3390/biology14020205/s1, Video S1: Comparative diagram of suitable and optimal spatial conditions by month for 2022.

## Appendix A

**Table A1:** The temperature range of Chinese water masses in winter and summer [34].

Water Mass		Winter (°C)	Summer (°C)
Bohai Sea, Yellow Sea, and East China Sea water masses	BY (Mixed Water of the BS and YS)	−1.0~2.0	25.0~27.0
	B_S_ (Coastal Water of the BS)	−1.0~2.0	23.0~27.0
	Y_S_ (Coastal Water of the YS)	2.0~4.0	24.0~25.0
	Y_C_ (YS Cold Water Mass)	-	6.0~15.0
	Y (YS Water)	2.0~9.0	22.0~27.0
	YE (Mixed Water of the YS and the ECS)	9.0~19.0	27.0~28.0
	E_F_ (Coastal Diluted Water of the ECS)	4.0~14.0	24.0~28.0
	E_S_ (Surface Water of the ECS)	13.0~17.0	27.0~29.0
	E (Mixed Water of the ECS)	13.0~17.0	-
	KS (Surface Water of Kuroshio)	20.0~25.0	26.0~30.0
	EK (Modified Surface Water of Kuroshio in the ECS)	15.0~20.0	-
	EU (Subsurface Water of the ECS)	-	14.0~22.0
	KU (Kuroshio Subsurface Water in the ECS)	-	16.0~23.0
	KM (Kuroshio Subsurface–Intermediate Mixed Water in the ECS)	12.0~16.0	12.0~16.0
	KI (Kuroshio Intermediate Water in the ECS)	5.0~12.0	5.0~12.0
	KD (Kuroshio Deep Water in the ECS)	<5.0	<5.0
South China Sea water mass	F (Diluted Water)	17.2~19.4	27.4~32.0
	M (Surface–Subsurface Mixed Water)	19.6~22.5	22.9~30.8
	S (SCS Surface Water)	21.0~29.5	22.3~31.9
	U_S_ (SCS Subsurface Water)	14.8~21.8	15.7~22.2
	UI (Subsurface–Intermediate Mixed Water)	10.5~14.9	11.0~15.6
	I (Intermediate Water)	5.3~10.4	5.5~11.1
	D (Deep Water)	2.4~5.2	2.4~5.5
	B (Bottom Water)	2.3~2.4	2.4~2.4
	KS (Kuroshio Surface Water)	19.5~26.3	25.4~29.7
	U_P_ (Northwest Pacific Subsurface Water)	19.4~20.4	15.9~24.9
Taiwan Strait Water	MZCW (Min-Zhe Coastal Water)	<14.0	23.1~25.2
	UW (Upwelling Water)	-	21.8~22.4
	NTMW (Northern Taiwan Mixed Water)	17.1~23.1	15.9~24.7
	ZJW (Zhujiang River Diluted Water)	-	27.6~29.1
	MCCW (Mixed China Coastal Water)	15.6~19.8	-
	SCSSW (SCS Surface Water)	17.3~20.8	25.7~30.5
	SCSW (SCS Water)	18.3~20.8	20.0~31.1
	KBW (Kuroshio Branch Water)	19.0~26.3	-
	SB-KBW (Subsurface Kuroshio Branch Water)	15.5~24.8	15.0~26.9
